# Omeprazole-induced galactorrhea in kidney transplant patients—a case report

**DOI:** 10.1186/s13256-022-03337-3

**Published:** 2022-03-27

**Authors:** Choki Dorji, Farruk Ahammed Robin, Kesara Na-Bangchang

**Affiliations:** 1 Pharmacovigilance Centre, Department of Pharmacy, Jigme Dorji Wangchuk National Referral Hospital, Thimphu, Bhutan; 2 Graduate Studies, Chulabhorn International College of Medicines, Thammasat University, Bangkok, Thailand; 3 Department of Medicine, Jigme Dorji Wangchuk National Referral Hospital, Thimphu, Bhutan

**Keywords:** Omeprazole adverse reaction, Omeprazole-induced galactorrhea, Omeprazole metabolism, CYP2C19, CYP3A4 interaction with tacrolimus

## Abstract

**Background:**

Omeprazole belongs to the pharmacological classifications of proton pump inhibitors and is a widely used medicine. All proton pump inhibitors have a common mechanism of action and are prodrugs that require activation in an acidic environment. Omeprazole is extensively metabolized in the liver by cytochrome 2C19 and cytochrome 3A4, which are responsible for drug interactions. Omeprazole-induced galactorrhea is a rare adverse event of drug metabolism and is often underreported.

**Case presentation:**

This is a case of a 26-year-old unmarried Asian (Bhutanese) female who underwent kidney transplant and was administered standard antirejection medication (tacrolimus, prednisolone, and leflunomide) along with an antihypertensive agent. She came to the emergency department with complaints of nausea, vomiting, abdominal pain, chronic gastritis, anemia, hypertension, and loss of appetite. The tacrolimus trough level was in the subtherapeutic range at admission. The tacrolimus dose was adjusted, and oral omeprazole was administered. After 3 days, she experienced milk production from her left breast, which according to the patient was her second incidence after omeprazole ingestion.

**Conclusion:**

Causality assessment using Naranjo’s algorithm and recovering from galactorrhea after stopping omeprazole and omeprazole rechallenge with the reappearance of galactorrhea confirmed omeprazole as the causative agent. Tacrolimus interferes with omeprazole metabolism and increases tacrolimus levels in the blood. Caution needs to be taken when omeprazole is administered with other drugs that interfere with metabolizing enzymes.

## Background

Omeprazole belongs to the pharmacological classifications of proton pump inhibitors (PPIs) and is a widely consumed medicine. All PPIs have a common mechanism of action and are prodrugs that require activation in an acidic environment. PPIs irreversibly inhibit the H^+^/K^+^ adenosine triphosphatase (ATPase) in gastric parietal cells, blocking acid production [1]. Omeprazole was the first approved PPI for public to use over the counter for the short-term management of heartburn [2–4]. In posttransplant patients, PPIs are prescribed to prevent gastric and peptic ulcer disease due to postsurgical stress and gastrointestinal side effects from mycophenolic acid and steroid use. Omeprazole is extensively metabolized in the liver by cytochrome 2C19 (CYP2C19) and cytochrome 3A4 (CYP3A4), causing it to interact with several other drugs. Omeprazole is safe for heartburn. The safety margin becomes narrow when omeprazole is used with drugs interfering with its metabolization. Omeprazole affects tacrolimus levels through a metabolic process that requires therapeutic drug monitoring of tacrolimus in recipients of kidney transplant to avoid acute graft rejection. Omeprazole-induced galactorrhea is a rare reaction based on the omeprazole metabolism process. Seven omeprazole-induced galactorrhea reactions have been reported in the VigiBase system maintained by Uppsala Drug Monitoring Centre as of 13 July 2021. Among these, two individual case safety reports (ICSRs) were reported from Germany and France, as well as one each from Spain, the Netherlands, and Bhutan. Often, rare drug reactions are unreported because such drug reactions are not labeled in the product summary characteristics. It is important to report a rare drug reaction in the form of a case study so that health professionals become aware of drug reactions.

## Case

**Fig. 1:**
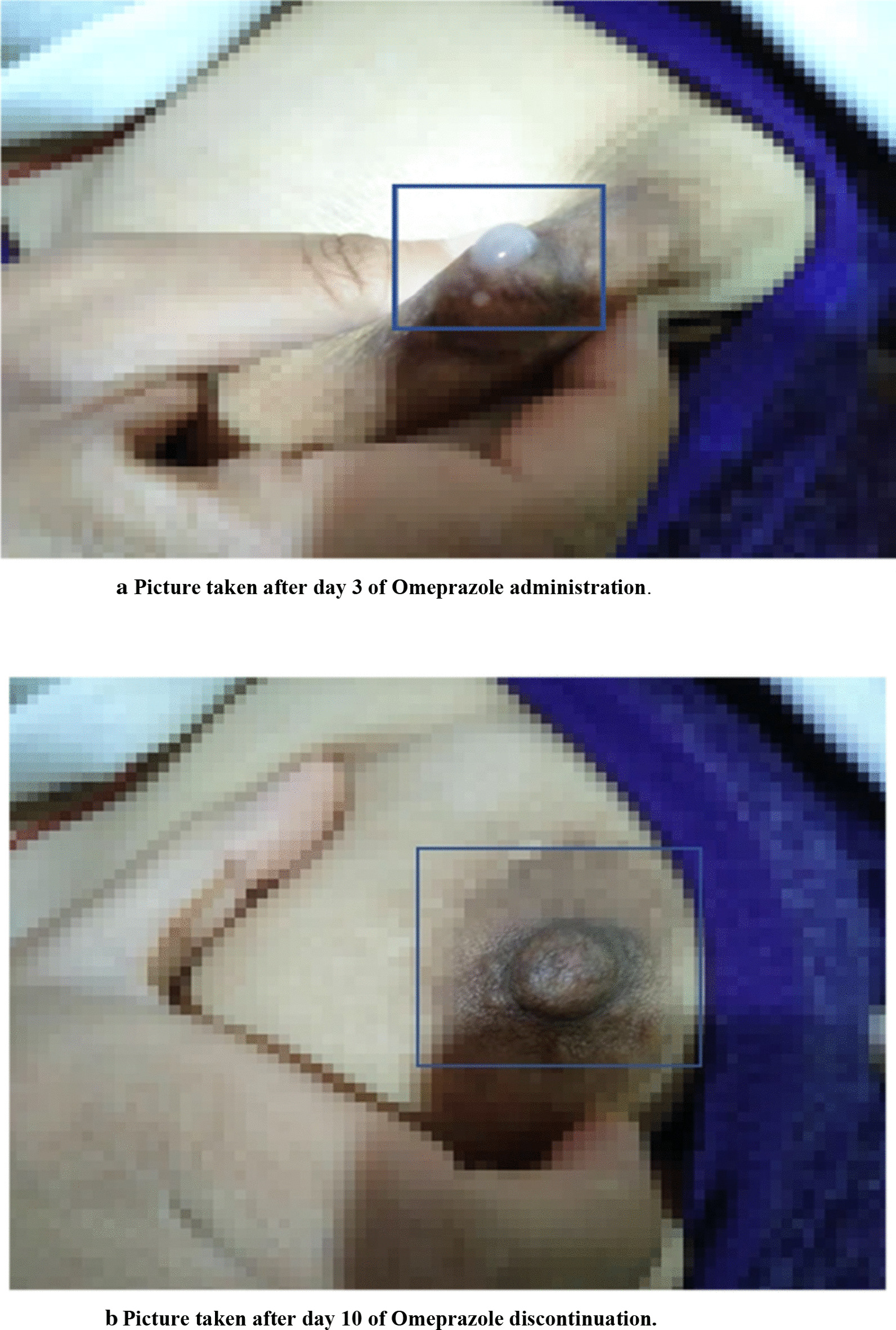
Omeprazole-induced galactorrhea

This is a case of 26-year-old unmarried, self-employed Asian (Bhutanese) woman, weighing 44 kg and 163 cm tall admitted to the medicine ward for nausea, vomiting, abdominal pain, chronic gastritis, anemia, hypertension, loss of appetite, and elevated serum blood urea and creatinine. The case was reported to the Pharmacovigilance Centre on 16 November 2020 as an adverse drug reaction. The patient underwent renal allograft replacement in 2013, and a second transplant was performed in 2015. Since then, she has been on regular oral antirejection and antihypertensive medication comprising tacrolimus (Tacrograf) 2 mg twice daily, prednisolone 5 mg once daily, leflunomide 20 mg once daily, nifedipine 40 mg twice daily, and hydralazine 50 mg three times daily. On the evening of 6 November 2020, she experienced nausea, vomiting, and severe abdominal pain and was brought to the emergency department, where physical examination was unremarkable, with no fever but a tender abdomen. Vital signs were as follows: blood pressure (BP) 152/94 mm/Hg, visual pain score 3/10, respiratory rate 18 breaths per minute; pulse rate 86 beats per minute; saturated partial oxygen (SpO_2_) 96%; and body temperature 96 °F. Laboratory findings were as follows: hemoglobin (Hb) 7.7 g/dl (11.3-14.9 g/dl); red blood cell (RBC) count 3.3 × 10^6^/μl (3.76–4.84 × 10^6^/μl); hematocrit (Hct) 24.7% (33–45%), white blood cell (WBC) count 21.4 × 10^3^/μl (4–10 × 10^3^/μl); serum creatinine (Cr) 8.6 mg/dl (0.6–1.2 mg/dl), and urea (Ur) 200 mg/dl (15–45 mg/dl). She received ceftriaxone 1 g intravenously once daily as an empirical antimicrobial therapy, paracetamol (acetaminophen) 300 mg intravenously three times daily, ranitidine 50 mg intravenously three times daily, thiamine 100 mg intravenously once daily, and intravenous infusion of lactate ringer with 5% dextrose in the emergency room along with antirejection medication. On 7 November 2020, she was transferred to the medicine ward. Her tacrolimus trough level was 3.07 ng/dl (4–8 ng/dl), Gravindex for urine sample was negative, 24-hour urine protein was 391 mg/dl (< 150 mg/dl), urine volume was 300 ml in 24 hours, and 24-hour protein was 1.7 g/24 hours (< 0.15 g/24 hours). The medication administered and laboratory reports in the medical ward are presented in Tables 1 and 2. After 3 days, on 10 November, the patient complained of milk production from a single breast (Fig. 1a). When interviewing the patient on her past medication use and the drug reaction, she revealed that she experienced a similar reaction to omeprazole in 2013 after kidney transplant. Oral omeprazole was immediately discontinued. The amount of milk production started to decrease, and on 18 November 2020, production stopped completely (Fig. 1b). Adverse drug reactions were entered into the Vigiflow system and reported to the National Pharmacovigilance Centre. The causality assessment score on Naranjo’s algorithm was 10 (> 9 Definite) (Table 3 and 4). On 20 November 2020, the tacrolimus trough level was 5.9 ng/dl (4–8 ng/dl) at a 2.5 mg twice daily dose. She was discharged from the hospital on 26 November 2020 and continued to receive treatment and dialysis as an outpatient.

**Table 1:** List of drugs administered in the medicine ward

Serial number	Name of concomitant drugs used	Start date	Remarks
1	Tacrolimus (Tacrograf) 2.5 mg BID PO	7 November 2020	Dose administered according to trough Co level. Medicine being continued
2	Leflunomide 20 mg OD PO	7 November 2020	No change in medicine since kidney transplant. Medicine being continued
3	Prednisolone 50 mg OD PO	7 November 2020	Dose increased from 5 mg OD to 50 mg OD on admission
4	Omeprazole 20 mg BID PO	7 November 2020	Discontinued on 10 November 2020 after suspecting ADE
5	Nifedipine 40 mg BID PO	7 November 2020	Medicine being continued with same dose as before
6	Hydralazine 50 mg TID PO	7 November 2020	Medicine being continued with same dose as before
7	Losartan 25 mg BID PO	14 November 2020	Medicine being continued and asked to follow up in the OPD
8	Furosemide 40 mg BID PO	15 November 2020	Medicine being continued and asked to follow up in the OPD
9	Valganciclovir 450 mg every 48 hours PO	14 November 2020	Empirical therapy for viral infection Dose adjusted according to serum creatinine level Medicine being continued and asked to follow up in the OPD
10	Ceftriaxone 1 g intravenous OD	6 November 2020	Empirical therapy for bacterial infection Dose adjusted according to serum creatinine level Discontinued on discharge
11	Fluconazole 150 mg OD PO	14 November 2020	Empirical therapy for fungal infection Help increase tacrolimus trough level Medicine being continued and asked to monitor tacrolimus drug level
12	Cotrimoxazole 480 mg OD PO	15 November 2020	Prophylaxes for pneumocystis pneumonia Medicine being continued and asked to follow up in the OPD
13	Sevelamer 800 mg TID PO	7 November 2020	Medicine being continued and asked to follow up in the OPD
14	Calcium 500 mg + vitamin D 250 IU TID PO	7 November 2020	Medicine being continued and asked to follow up in the OPD
15	Thiamine 75 mg OD PO	7 November 2020	Medicine being continued and asked to follow up in the OPD
16	Sodium bicarbonate 500 mg TID PO	14 November 2020	Medicine being continued and asked to follow up in the OPD
17	Vitamin C 250 mg TID PO	13 November 2020	Medicine being continued and asked to follow up in the OPD
18	Potassium chloride 600 mg TID PO	14 November 2020	Medicine being continued and asked to follow up in the OPD

*BID* twice daily, *OD* once daily, *TID* thrice daily, *IU* international unit, *PO* per oral route

**Table 2:** Laboratory parameters from date of admission to discharge from hospital

Laboratory parameters	7 November 2020	15 November 2020	23 November 2020
White blood cell (4–10 × 10^3^/μl)	21.4	26.7	18.5
Red blood cells (3.76–4.84 × 10^3^/μl)	3.3	3.9	3.3
Hematocrit (33–45%)	24.7	31.5	27
Hemoglobin (11.3–14.9 g/dl)	7.7	9.6	8.2
Platelets (150–450 × 10^3^/μl)	208	247	207
Urea (15–45 mg/dl)	200	100	30
Creatinine (0.6–1.2 mg/dl)	8.6	6.1	2.2
Sodium (133–146 mEq/L)	140	136	134
Potassium (3.8–5.4 mEq/L)	3.1	2.9	3.6
Chloride (96–110 mEq/L)	107	112	105
SGOT (AST) (5–40 IU/L)	22	18	16
ALT (5–40 IU/L)	16	32	18
Alkaline phosphatase (35–104 IU/L)	130	114	110
Total bilirubin (0.1–1.2 mg/dL)	0.3	0.2	0.2
Direct bilirubin (< 0.2 mg/dL)	0.1	0.1	0.1
Tacrolimus (4–8 ng/ml)	3.07	–	5.9

*ALT* alanine transaminase,* SGOT(AST)* serum glutamic-oxaloacetic transaminase (aspartate aminotransferase),* 103/uL* cells per microliter,* g/dL* gram per deciliter,* mg/dL* milligram per deciliter,* mEq/L* milliequivalents per Liter,* IU/L* international unit per liter,* ng/mL* nanogram per milliliter

**Table 3.** Causality assessment using Naranjo’s algorithm

**Question**	**Yes**	**No**	**Don’t know**	**Score**
1. Are there previous conclusive reports on this reaction?	+1	0	0	1
2. Did the ADR appear after the suspected drug was administered?	+2	− 1	0	2
3. Did the ADR improve when the drug was discontinued?	+2	0	0	2
4. Did the ADR appear with rechallenge?	+2	− 1	0	2
5. Are there alternative causes for the ADR?	− 1	+2	0	2
6. Did the reaction appear when placebo was given?	− 1	+2	0	0
7. Was the drug detected in blood at toxic levels?	+1	0	0	0
8. Was the reaction more severe when the dose was increased or less severe when the dose was decreased?	+1	0	0	0
9. Did the patient have a similar reaction to the same or similar drug in any previous exposure?	+1	0	0	1
10. Was the ADR confirmed by any objective evidence?	+1	0	0	0
Total score				10

Total score	Check one □
≥ 9	**√** Definite
5–8	□ Probable
1–4	□ Possible
≤ 0	□ Doubtful

The Definite score correlates: followed a reasonable temporal sequence after a drug or in which a toxic drug level had been established in body fluids or tissues; followed a recognized response to the suspected drug, was confirmed by improvement in withdrawal of the drug and reappearance upon re-exposure

*ADR* adverse drug reaction

## Discussion

Galactorrhea is defined as nonlactational milk production at 1 year after pregnancy and cessation of breastfeeding. It can occur in nulliparous and postmenopausal women and even in men. Although the incidence varies, it can occur in 90% of women with hyperprolactinemia. Estrogen, progesterone, and prolactin (PRL) are essential for breast development and lactation. Drug-induced hyperprolactinemia is a concern of underreporting drug reactions due to a lack of externally visible symptoms, patient reluctance due to embarrassment, and lack of awareness among clinicians. In a pharmacoepidemiological analysis of the French pharmacovigilance database from 1985 to 2000, of 182,836 spontaneous adverse drug reaction reports, 159 (0.08%) drug reactions were related to hyperprolactinemia. The male-to-female sex ratio was 5.9 (136 women and 29 men), and the mean age was 40 (range 14–85) years. In the same study, the class of drugs reported included neuroleptics (31%), neuroleptic-like drugs (28%), antidepressants (26%), H2 receptor antagonists (5%), and others (10%). All of these drugs inducing galactorrhea were reported only in the literature and were not labeled in the product summary characteristics [5, 6]

The mechanism by which neuroleptic drugs induce hyperprolactinemia involves regulation of dopamine and PRL in the brain. Four dopaminergic pathways involved are as follows: (i) mesolimbic tract, (ii) mesocortical tract, (iii) nigrostriatal tract, and (iv) tuberoinfundibular dopaminergic (TIDA) tract. Increased PRL results in higher activity of TIDA neurons, whereas a decrease in circulating PRL levels lowers their activity. Dopamine, which is released from the terminal in the median eminence of the hypothalamus, travels down the pituitary through the portal veins, and PRL controls its release by altering release through a mechanism called “short-loop feedback regulation.” As the level of PRL increases, the amount of dopamine available to the pituitary increases. Dopamine is a predominant inhibitor of PRL secretion. Released dopamine binds to the dopaminergic 2 receptor (D_2_R) on the membrane of lactotroph cells and inhibits PRL gene transcription and proliferation of lactotrophs, which further inhibits PRL synthesis and release [7, 8]. Elevated estrogen levels cause hyperplasia of lactotrophs and hyperprolactinemia by antidopaminergic action at the pituitary level [9, 10].

The mechanism by which antidepressants may cause hyperprolactinemia is not well understood, though several theories explain the involvement of serotonin stimulation of GABAergic neurons and indirect modulation of prolactin release. A role of serotonin in the elevation of PRL has been proposed [10–13]. PRL is synthesized and secreted from the anterior pituitary gland. In pathophysiological conditions such as thyroid disorder, vasoactive intestinal polypeptide stimulates PRL synthesis and thyrotropin-releasing hormone increases secretion; in renal impairment, it decreases PRL clearance and increases serum PRL levels. [14, 15].

Drugs including antihypertensive drugs (verapamil, methyldopa) and gastrointestinal motility drugs (metoclopramide) are reported to cause hyperprolactinemia. Verapamil is believed to cause hyperprolactinemia by blocking hypothalamic generation of dopamine and methyldopa by inhibiting the enzyme aromatic-l-amino decarboxylase, which is responsible for converting l-dopa to dopamine. Metoclopramide and domperidone are dopamine receptor blockers. More than 50% of patients taking these drugs experience effects, including amenorrhea and galactorrhea in women and impotence in men [9, 14, 16, 17]. A study involving metoclopramide-treated patients reported an average length of time from the end of treatment to disappearance of galactorrhea at 57 (± 12) days [16]. However, our patient had not taken any of the mentioned drugs at or prior to admission. Her regular medication includes antirejection and antihypertensive drugs excluding verapamil or methyldopa. In the emergency room, she received two doses of injection ranitidine. There are few cases in which galactorrhea is reported to be due to cimetidine and ranitidine; however, in most of these cases, the drug reaction occurred after 30–60 days of administration [18–20]. In our case, we did not suspect ranitidine as the culprit drug considering the dose and duration of injection she received on admission.

The level of PRL is usually raised with symptoms of galactorrhea [14]. However, the test was not performed for our patient, as the PRL test is rarely advised in our hospital. According to verbal information, the patient experienced a similar reaction to omeprazole in 2013 when she was undergoing kidney transplant in India and recovered after discontinuing omeprazole. Documentation of her past medical history, including records on drug reactions, was not available when she was admitted.

There are reports of cases similar to the present one in recipients of kidney transplant who had elevated serum PRL levels at 140 ng/mL (2.8–29.2 ng/mL). Two weeks after stopping omeprazole, serum PRL levels returned to normal (18.8 ng/mL), accompanied by resolution of galactorrhea [21].

In another case of rechallenge, esomeprazole-induced galactorrhea was associated with elevated fasting prolactin levels of 276 ng/ml (5–25 ng/ml) and 656 pg/ml (20–145 pg/ml) estradiol after esomeprazole administration for 7 days. Magnetic resonance imaging (MRI) brain and thyroid function tests were normal, and a urine pregnancy test negative. Upon discontinuing esomeprazole for 3 days, galactorrhea was resolved, and PRL levels declined to 23 ng/ml. After 7 days, the estradiol level also returned to normal. After 1 month, the patient took esomeprazole again (rechallenged) and came to the OPD with galactorrhea [22].

Adverse reactions from drug interactions (DIs) have been reported for PPIs when co-administered with drugs that are metabolized by the CYP2C19 and CYP3A4 enzymes. Omeprazole is a prodrug and needs to undergo metabolism for its acid inhibitory action. Hydroxy-omeprazole and omeprazole sulfone are two metabolites of omeprazole formed by CYP2C19 and CYP3A4, respectively [23, 24]. PPIs are frequently prescribed for kidney transplant patients after surgery to overcome steroid-induced gastritis. Antirejection drugs, especially calcineurin inhibitors (tacrolimus and cyclosporine), interact with omeprazole in recipients of kidney transplant who have CYP2C19 gene mutations and use CYP3A4 for intestinal and hepatic elimination, resulting in increased tacrolimus levels [25]. Omeprazole, lansoprazole, and pantoprazole are sensitive to degradation in the stomach acidic medium. Therefore, they are administered in modified formulation to overcome this barrier. Omeprazole and lansoprazole are formulated as enteric-coated granules in hard gelatin capsules, and pantoprazole is formulated as an enteric-coated tablet [23]. Pantoprazole shows a stronger inhibitory effect on CYP3A4, followed by omeprazole, esomeprazole, rabeprazole, and lansoprazole [2].

The drug metabolism process contributes to the mechanism by which omeprazole induces galactorrhea by inhibiting CYP3A4, leading to decreased metabolism of estrogen and thereby increasing serum estrogen levels. When estrogen is stimulated, prolactin is released by increasing mitotic activity in pituitary lactotrophs, enhancing prolactin gene transcription indirectly through vasoactive intestinal peptide and oxytocin gene expression (22, 26, 27). Drug substrates such as tacrolimus administered with omeprazole or fluconazole (CYP2C19 and CYP3A4 inhibitor) will increase the tacrolimus level [2]. In our patient, the tacrolimus level was 5.9 ng/dl (4–8 ng/dl) on 20 November 2020 after administering oral fluconazole. Impaired renal function and accumulation of omeprazole metabolites may have triggered CYP3A4 inhibition. As cytochrome 3A4 metabolizes estrogen, estrogen levels increase when the enzyme is inhibited. Elevated estrogen levels are responsible for hyperprolactinemia.

Naranjo’s algorithm was used to assess the causal relationship between the suspected drug and drug reaction. The causality assessment established the (i) temporal relationship between drug and drug reaction, (ii) biological plausibility, (iii) dechallenge, and (iv) rechallenge. Our patient was given omeprazole and developed galactorrhea within 3 days. As the patient developed a suspected drug reaction, omeprazole was discontinued. The patient started recovering after stopping omeprazole and was successfully dechallenged. The patient had a history of galactorrhea induced by omeprazole, and the current onset of galactorrhea after omeprazole administration confirmed successful rechallenge. As galactorrhea started after omeprazole was initiated, there was a clear-cut temporal correlation of omeprazole with omeprazole-induced galactorrhea. To the best of our knowledge, it is biologically plausible that omeprazole can cause galactorrhea through the mechanism mentioned above. Thus, considering the above cardinal aspects of causality of adverse drug reaction and Naranjo’s algorithm, we confirmed this reaction to be definite, with a score of 10. This event has been reported to the National Pharmacovigilance Centre and to the WHO-UMC International Drug Monitoring Centre via the Vigiflow system. The past history of our patient offered solid clues to suspect omeprazole as the culprit drug; however, owing to lack of documentation and laboratory evidence, more studies are needed.

## Conclusion

After causality assessment using Naranjo’s algorithm, the patient recovered from galactorrhea after stopping omeprazole; self-rechallenge by the patient herself with reappearance of galactorrhea helped to confirm omeprazole as the causative agent. Tacrolimus interferes with omeprazole metabolism and increases tacrolimus levels in the blood. Caution needs to be taken when omeprazole is administered with other drugs that interfere with metabolizing enzymes.

## Data Availability

Figure 1 and Tables 1, 2, 3,

**Table 4:** Previously published case report on PPI-induced galactorrhea

Age/sex	PPI	Dose	Duration between drug intake and galactorrhea	Serum PRL level (ng/ml)	Refs.
13 years old/female	Omeprazole	Not reported	4 days	288	[28]
	Lansoprazole		7 days	49.7	
32 years old/female	Esomeprazole	40 mg daily	7 days	276	[22]
21 years old/male	Lansoprazole	15 mg daily	265 days	32	[29]
26 years old/female	Omeprazole	40 mg twice daily	90 days	140	(21)
26 years old/female	Omeprazole	20 mg twice daily	4 days	Test missed	In our case

and 4 attached. Previously published case report on PPI-induced galactorrhea

## Abbreviations

ADR: Adverse drug reaction
ATPase: Adenosine triphosphatase
BP: Blood pressure
CYP2C19: Cytochrome 2C19
CYP3A4: Cytochrome 3A4
Cr: Creatinine
MRI: Magnetic resonance imaging
OPD: Out-patient department
PRL: Prolactin
PPIs: Proton pump inhibitors
RBC: Red blood cell
SpO_2_: Saturated partial oxygen
TRH: Thyrotropin-releasing hormone
UPT: Urine pregnancy test
Ur: Urea
UMC: Uppsala Monitoring Centre
VIP: Vasoactive intestinal polypeptide
WBC: White blood cell
